# Experiments and Observations on Land and Sea Air

**Published:** 1801-09

**Authors:** Adam Seybert


The importance of Eudiometrical Obſervations and Experiments, as leading to an improvement of the means of preventing diſeaſe, has long been acknowledged: In continuation, therefore, of the ſubject commenced at page 10, of the preſent volume, we extract from the American Philoſophical Tranſactions, No. XXXII. the following

### *Experiments and Observations on Land and Sea Air.* By Adam Seybert, M. D.

An endeavour to add any facts or obſervations to a branch of knowledge, which has been treated of by many of the moſt enlightened philoſophers of the preſent century, may be deemed a hazardous attempt. But although we have many accounts of eudiometrical experiments by Prieftley, Fontana, Ingenhoufz, and others, the fubject is not exhaufted, and an extenfive field continues open for him who wifhes to engage in this intricate branch of pneumatic philofophy.

The purity of the air is not interefting to us merely as an object of curiofity, but demands our attention as phyficians and philofophers. In proportion to the number of afcertained facts, the certainty of inference is increafed. The fhort life of any one individual, together with his local fituation, will prevent him from completing this department of fcience. It is merely from repeated experiments made under different circumftances, that we can expect to arrive at truth. The more we multiply facts, the more decided may we be in our conclufions. Such are the reflections, which induced me to engage in a feries of experiments, which fhall be related in the following pages.

Our atmofphere having been fo fuccefsfully analyzed by the celebrated Lavoifier, and being found to confift of fluids poffeffing very different and oppofite qualities, chemifts foon began to enquire whether its ingredients might not be in various proportions in different fituations; and, particularly, whether it differed in point of purity in different fituations on land and on the ocean.

Moft of the experiments, of which we have an account, were made on land: The Memoir of Dr. Ingenhoufz, publifhed in the 70th volume of the Philofophical Tranfactions, is the only effay I have feen containing experiments made at fea; but his traverfe was fo fhort, that he had not an opportunity of examining the air in different latitudes. He, however, is of opinion, that fea air is, cæteris paribus, purer than land air; but he appears to have found fome feeming contradictions of his general inference. He fays, page 364, that air taken from the middle of the channel was of an inferior quality to that at the mouth of the Thames; and that air near the fea fhore at Oftend, was nearly as good as that at the mouth of that river. Although we may, to a great degree, adopt his fentiments, neverthelefs, I think it probable, that this increafed purity does not entirely depend upon the ocean; for I have found the air over the bays of Chefapeak and Delaware of the fame degree of purity with the atmofphere of the ocean. And hence I am inclined to think, that the air over a large body of water, is always purer, cæteris paribus, than that of the adjoining land, owing perhaps to a decompofition, which the water may fuffer from the action of the fun's rays; and this may likewife be affifted by its alfo abforbing many foreign matters which which on land are more or lefs intimately mixed with the air in a mechanical way. This opinion is confirmed by Dr. White's experiments, who fays, "the air over the river Ouze was conftantly purer than that of the garden by two or three degrees." Philofophical Tranfactions, vol. 68. And in the fame paper he obferves, that the fame happened with the air of the fofs when the marfhes were overflowed.

When I firft engaged in thefe experiments, it was my intention to perform them only on fea air; but I foon found it neceffary to repeat them on land air, for the fake of comparifon. The fubject increafed on my hands: The atmofphere of marfhes prefented itfelf as worthy of ferious inveftigation; I therefore performed fome experiments upon it: but proper length of time is neceffary to their repetition, and for this reafon I muft omit them for the prefent, and merely relate thofe I performed on the air of this city, its environs, and on the ocean.

I fhall firft proceed to the enquiry, whether the atmofphere differs in purity in different fituations on land?

The opinion that the air is purer in the country and on the tops of mountains than it is in towns, is adopted by many; therefore, in afferting the contrary, we muft prepare to meet with oppofition, particularly from thofe who have formed opinions from reafoning alone, unfupported by experiments. In doubtful matters, it is chiefly by the clafhing of opinion that truth is finally difcovered. This fhall be both my confolation and apology, if the refult of my experiments fhall be found to have induced me to differ from others. Neverthelefs, it will afford me confiderable fatisfaction to agree with thofe whofe decifions reft upon the fame firm bafis; I fhall, therefore, briefly mention the authors who agree with me in opinion.

Dr. Prieftley concludes from his own experiments, that the difference of the air in different places, fuch as is indicated by a mixture of nitrous air, is in general very inconfiderable. He mentions, that the air of Harthill, near Manchefter, and that of Wiltfhire, were about the fame.

The compilers of the Encyclopædia fay, "that the general mafs remains upon all occafions pretty much the fame." And Scheele is much of the fame opinion.

But the accurate Fontana fpeaks with more confidence, and is more explicit. His affertions are founded upon the refult of many experiments, and he is inclined to believe, that the flight variations mentioned by fome philofophers, are rather to be attributed "to the fallacious effects of uncertain methods," than to any real difference in the air itfelf. He found the air of Iflington and London to fuffer an equal diminution from the mixture with nitrous air. Air taken at different heights in London

London and Paris did not differ in purity Air at the height of 313 and 202 feet in London, differed ſcarcely at all; and no difference was perceptible between the air of theſe heights and that of the ſtreet adjoining.

The more I reflect on this ſubject, the more I am inclined to adopt the following ſentiment of this laſt mentioned gentleman, viz. "The difference in the purity of the air at different times, is much greater than the difference between the air of the different places." Indeed, moſt of the experiments related by Dr. Ingenhouſz alſo tend to confirm it. In general, the difference in the air of different places at the ſame time was by no means conſiderable.

I ſhall now with more confidence relate the experiments I myſelf performed; but previous to this recital I ſhall give a brief account of the method I purſued.

It is neceſſary to remark, that every experiment I ſhall relate is the reſult of, at leaſt, two different trials.

Moſt authors who have engaged on this ſubject uſed eudiometers of a different conſtruction; I adopted the moſt ſimple as the beſt. Thoſe who deſire a particular deſcription of theſe inſtruments, may be ſatisfied by referring to the Encyclopædia, and different parts of Dr. Prieſtley's Treatiſe on Air. Mine is as follows:*

I had a glaſs tube about fourteen inches in length, and in diameter nearly half an inch, provided with a graduated ſcale, made ſo as to ſlide upon the tube, up or down, as occaſion required. This ſcale was divided into one hundred equal parts.

My meaſure was a ſmall ſmelling bottle, containing ʒj. and gr. xvj. of clear pump water. The ſpace occupied in the tube by a bulk of air which this meaſure could contain, was equal to the hundred diviſions of the graduated ſcale.

My water trough on board of the ſhip was the common water bucket; on ſhore, it was a common houſe bucket or tub.

The

---

* The atmoſphere is proved by inconteſtable experiments to conſiſt in general of,

Oxygen gas, 0,27.

Azotic gas, 0,72; and

Carbonic Acid, 0,01.

It is a fact well known to chemiſts, that nitrous air will combine with oxygen gas, and form a compound, viz. the nitric acid. As theſe two gaſes combine, they aſſume a ſtate approaching nearer to that of a ſolid, and conſequently occupy leſs ſpace than they did before their union. Upon this diminution of bulk depends our eſtimation of the purity of the air: The greater the contraction, the purer we ſuppoſe the air under trial.

The nitrous gas was prepared from diluted nitric acid and brafs filings.

At fea, I ufed fea water in the trough; on land, common pump water; for from different trials made by Dr. Ingenhousz, it is evident this circumftance could not produce a variation in the refult of the experiments.

My method of operating is as follows: After having introduced two meafures of the air, whofe purity I defired to afcertain, into the glafs tube, I introduce one meafure of nitrous gas; then, fuffering the tube to remain undifturbed for about a minute, I noted down how far the water afcended without agitation; this is what I have called, upon mixture: I then agitated the tube three fucceffive times, after the manner of M. de Sauffure, and noted how high the water rofe. In many inftances I added a fecond meafure of nitrous gas, and thereby completely faturated the air under examination.

I was particularly cautious of avoiding miftakes from hurry or inattention, and took fome pains to guard againft all the circumftances Dr. Ingenhoufz mentions as liable to produce a variation in the refult of experiments of this kind.

My firft experiment on land air was performed Auguft 2, 1796. Two meafures of air in the yard of my lodging, when mixed with one meafure of nitrous air, left upon mixture 2.48 of a meafure; and after fhaking the tube 1.79. I then added another meafure of nitrous air and 2.65 remained.

I then fubmitted air to the teft of the eudiometer, which I had previoufly collected in different ftreets of this city, viz. in Water between Market and Arch Streets; in Spruce near Fourth Street; in Chefnut near Fifth; and, in Market between Second and Third Streets. Each of thefe airs gave nearly the fame refult, and generally agreed with that of the air of my lodging: None of the experiments fhew a difference of 0.02 of a meafure.

Similar experiments I have fince repeated and the refult was the fame.

Auguft 3d. I collected air on the top of the hill whereupon Dr. Smith's Obfervatory ftands, at the Falls of Schuylkill, five miles from Philadelphia. In another vial I received air from above the middle of the road directly at the foot of the hill. And immediately on my return home I fubmitted them and the air of the yard to experiment, and found them to agree exactly as follows

Upon mixture 2.48.

After fhaking the tube 1.78 and upon adding a fecond meafure of nitrous air, 2.63 remained.

Auguft

Auguſt 5th. I collected air from above two different marſhy ſituations immediately below the rope-walks to the ſouth of this city. It is of conſequence to remark that theſe marſhes are overflowed by the tide. Another vial I filled immediately before entering the city in Front Street. Theſe airs ſuffered an equal diminution from a mixture of nitrous gas, viz. 2.47 upon mixture; after ſhaking the tube 1.79; and after adding a ſecond meaſure of nitrous gas 2.64 remained.

The air near my lodging yielded upon mixture 2.49; after ſhaking the tube 1.78; and upon the addition of a ſecond meaſure of nitrous gas 2.62.

I performed ſome experiments on air collected in other ſituations about the city; but finding the reſult ſo much the ſame as thoſe above related, I did not make any note of them, and remain perfectly ſatisfied that M. Fontana's aſſertion is well founded.

To theſe experiments I will ſubjoin thoſe I made on the ocean during a paſſage from Bourdeaux to Philadelphia. It appeared to me preferable to connect them in the form of a table, as thereby I ſhould avoid a needleſs repetition; and place before the reader a ſhort though accurate view of all the experiments at the ſame time.

The experiments I performed on the river Elk and bay of Cheſapeak perfectly agree with each other; and the reſult was the ſame as thoſe performed on the 7th of July, &c. The wind blew from the north, and the ſky was partially cloudy. They were performed in Auguſt laſt.

My experiments at ſea ſufficiently prove that the atmoſphere is conſiderably purer there than it is on land. Though there are ſome trifling differences in the reſults of ſeveral experiments, I have no reaſon to believe that they were owing to the different ſituation in point of latitude or longitude in which they were performed. I can form no ſyſtem reſpecting ſuch variations. Winds, temperature, rain, &c. do not ſeem to have produced them. As they did not obſerve any regularity in their occurrence, they may perhaps be attributed to certain unperceived errors which are unavoidably attendant on ſuch trials.

[ To be continued. ]

